# *Chlamydia pneumoniae* infection and inflammasome activation in Alzheimer’s retina: Correlations with disease status

**DOI:** 10.1002/alz.087842

**Published:** 2025-01-03

**Authors:** Bhakta Prasad Gaire, Yosef Koronyo, Lalita Subedi, Altan Rentsendorj, Dieu‐Trang Fuchs, Natalie Swerdlow, Keith L Black, Moshe Arditi, Timothy R Crother, Maya Koronyo‐Hamaoui

**Affiliations:** ^1^ Department of Neurosurgery, Maxine Dunitz Neurosurgical Research Institute, Cedars‐Sinai Medical Center, Los Angeles, CA USA; ^2^ Department of Pediatrics, Division of Infectious Diseases and Immunology, Cedars‐Sinai Medical Center, Los Angeles, CA USA; ^3^ Department of Biomedical Sciences, Infectious and Immunologic Diseases Research Center, Cedars‐Sinai Medical Center, Los Angeles, CA USA; ^4^ Department of Neurology, Cedars‐Sinai Medical Center, Los Angeles, CA USA; ^5^ Department of Biomedical Sciences, Division of Applied Cell Biology and Physiology, Cedars‐Sinai Medical Center, Los Angeles, CA USA

## Abstract

**Background:**

An emerging theory suggests a link between Alzheimer’s disease (AD) and microbial infection. Notably, various microbes have been detected in the post‐mortem brains of AD patients and murine models. However, there exists a gap in research concerning the presence and role of microbial infection in the AD retina, which shares common pathogenesis with the brain. Focusing on *Chlamydia pneumoniae* (Cp), a gram‐negative respiratory pathogen, we assessed the presence and severity of infection and inflammasome activation in retinas and paired brains from individuals with mild cognitive impairment (MCI) or AD, comparing them to age‐ and sex‐matched counterparts with normal cognition (NC). This investigation provides critical insights into the potential microbial involvement in AD pathology.

**Method:**

Employing diverse histological and biochemical techniques, we identified and quantified Cp and related biomarkers in retinas and brains of MCI and AD patients vs NC controls. We determined the relationships between retinal and brain Cp infection and gliosis, inflammasome activation, pyroptosis, and disease status.

**Result:**

Cp inclusions were identified in the postmortem retina of individuals with MCI and AD, correlating with Cp burden in the corresponding brain tissues. Notably, a threefold increase in retinal and brain Cp infection was observed in AD, not in MCI, compared to NC, suggesting a predilection of Cp infection at later AD stages. Live Cp was successfully isolated from AD retinal lysates, confirming active Cp infection. Retinal Cp infection correlated with retinal amyloidosis and synaptic damage, but not with brain amyloidosis. Additionally, it was associated with brain tauopathy markers as well as the cognitive status. Cp‐infected cells were engulfed by retinal microglia. Furthermore, Cp infection triggered NLRP3‐inflammasome activation, gliosis, pyroptosis, and apoptosis. While retinal NLRP3 inflammasome increased earlier in MCI, Cp‐induced retinal ASC, Capase‐1 and ‐3, and cleaved‐GSDMD rose later in AD‐dementia stage. These findings unveil the intricate relationship between Cp infection, retinal pathology, and AD progression.

**Conclusion:**

Our findings reveal Cp infection in both AD retina and brain, coupled with inflammasome markers. This highlights a potential link to neurodegeneration in AD, offering avenues for therapeutic exploration, involving early antibiotic treatment or inflammasome attenuation.

